# New Sanatoria

**Published:** 1921-02-12

**Authors:** 


### New Sanatoria.

A new sanatorium for Leeds is proposed on the site of 118 acres in King Lane, Moortown, lately transferred by the Improvements Committee to the Leeds Health Committee.

---

The West Bromwich Town Council has approved the purchase of the Red House, Great Barr, for use as a sanatorium for children and adults. The estimated cost of the estate, buildings, alterations, and equipment works out at £292 per bed; there are about seventy in all. After allowing for the Government grants, £4,382 is expected to fall on the rates.

